# Reinforcing Bulk Heterojunction Morphology through Side Chain-Engineered Pyrrolopyrrole-1,3-dione Polymeric Donors for Nonfullerene Organic Solar Cells

**DOI:** 10.1021/acsaem.4c02670

**Published:** 2025-01-07

**Authors:** Danbi Kim, Vellaiappillai Tamilavan, Chieh-Szu Huang, Yang Lu, Eunhye Yang, Insoo Shin, Hyun-Seock Yang, Sung Heum Park, Samuel D. Stranks, Bo Ram Lee

**Affiliations:** † School of Advanced Materials Science and Engineering, Sungkyunkwan University (SKKU), Suwon 16419, Republic of Korea; ‡ Department of Chemical Engineering and Biotechnology, University of Cambridge, West Cambridge Site, Philippa Fawcett Drive, Cambridge CB3 0AS, United Kingdom; § Cavendish Laboratory, University of Cambridge, J. J. Thomson Avenue, Cambridge CB3 0HE, United Kingdom; ∥ Department of Physics, Pukyong National University, Busan 48513, Republic of Korea; ⊥ Department of Chemical and Biomolecular Engineering, New York University Tandon School of Engineering, Brooklyn, New York 11201, United States

**Keywords:** wide band gap polymer, halogen substituent effect, morphology, solid additive, polymer solar cells

## Abstract

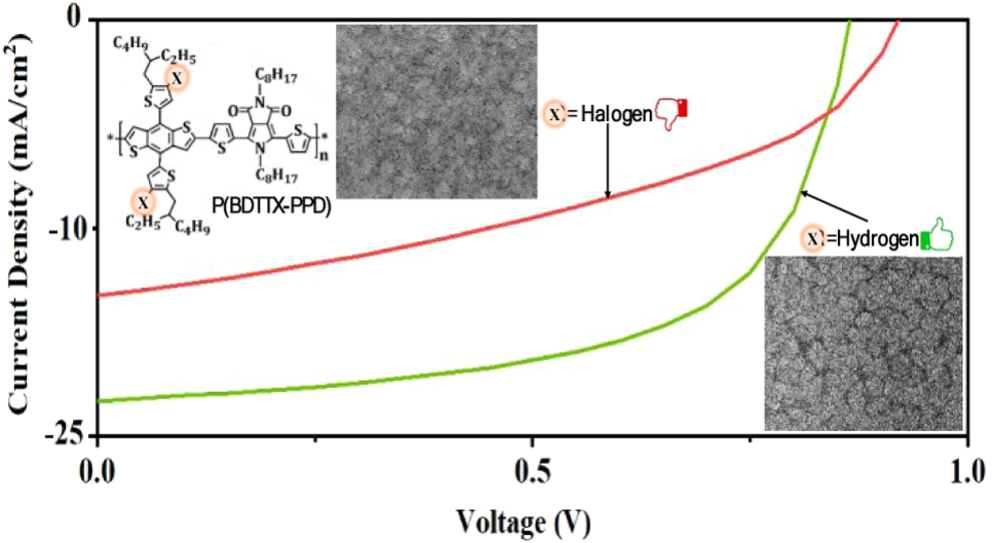

Organic solar cells (OSCs) are attracting significant attention due to their low cost, lightweight, and flexible nature. The introduction of nonfullerene acceptors (NFAs) has propelled OSC development into a transformative era. However, the limited availability of wide band gap polymer donors for NFAs poses a critical challenge, hindering further advancements. This study examines the role of developed wide band gap halogenated pyrrolo[3,4-*c*]pyrrole-1,3(2H,5H)-dione (PPD)-based polymers, in combination with the Y6 nonfullerene acceptor, in bulk heterojunction (BHJ) OSCs. We first focus on the electronic and absorbance modifications brought about by halogen substitution in PPD-based polymers, revealing how these adjustments influence the HOMO/LUMO energy levels and, subsequently, photovoltaic performance. Despite the increased *V*_oc_ of halogenated polymers due to the optimal band alignment, power conversion efficiencies (PCEs) were decreased due to suboptimal blend morphologies. We second implemented PPD as a solid additive to PM6:Y6, forming ternary OSCs and further improving the PCE. The study provides a nuanced understanding of the interplay between molecular design, device morphology, and OSC performance and opens insights for future research to achieve an optimal balance between band alignment and favorable blend morphology for high-efficiency OSCs.

## Introduction

In the realm of photovoltaic research, organic solar cells (OSCs) are garnering significant interest for their inherent advantages, which include flexibility, lightweight nature, and the capacity for economical large-scale manufacturing.^1−5^ These traits make them exceptionally suitable for integration into portable electronic devices.^6,7^ The bulk heterojunction (BHJ) structure, which incorporates a blend of donor and acceptor materials within the active layer, is becoming increasingly prominent owing to its high interfaces yielding superior efficiency.^1,8^ Under irradiation, excitons are generated within these donor and acceptor materials. These excitons are then driven to the interface, where they are dissociated into free charge carriers that are subsequently collected by the electrodes.^9^ The efficacy of this exciton dissociation and charge extraction is highly contingent upon the energy level alignment between the donor and acceptor materials.^10−12^ Minimizing the energy differential between the Highest Occupied Molecular Orbital (HOMO) levels and the Lowest Unoccupied Molecular Orbital (LUMO) levels of the donor and acceptor materials is critical, as it reduces energy loss (*E*_loss_) during exciton dissociation.^13−15^ Therefore, fine-tuning the energy levels of donor and acceptor materials is a crucial aspect of optimizing BHJ OSCs.

It is well-established that incorporating electron-withdrawing elements, e.g., halogens, into a molecule can significantly alter its electronic properties.^16−18^ Specifically, these elements can deepen the HOMO–LUMO levels due to a “push–pull” effect which influences the electronic distribution and the optical and electronic properties of the molecule.^19,20^ This principle has been effectively utilized in designing donor/acceptor materials with matched HOMO–LUMO levels, leading to a higher open-circuit voltage (*V*_oc_). For instance, PBDBT has been engineered with substituents like hydrogen (H), fluorine (F), and chlorine (Cl) at specific sites on its molecular moiety.^21^ Particularly, the introduction of F and Cl has been found to better align the HOMO–LUMO levels with the Y6 acceptor, thereby enhancing the *V*_oc_.^22^ Halogen substitutions like fluorine and chlorine, despite their small van der Waals radius and high electronegativity, lower the HOMO levels of the resulting polymers, leading to improved energy alignment with acceptors.^23,24^ Furthermore, halogen substitutions enhance absorption around 400–500 nm and promote stronger intermolecular packing via π–π interactions between aromatic side chains, contributing to improved film morphology and device performance.^25^ Substituting side elements in molecular structures not only modifies their electronic properties but also influences other crucial aspects such as blend compatibility, interfacial characteristics, bulk heterojunction (BHJ) morphology and, consequently, the overall performance of the device.^26^ While previous studies on elemental substitution have primarily focused on energy-level alignment and interactions within halogen groups,^22,27^ there has been relatively less discussion about how such substitutions affect the intermixing of donor and acceptor materials and the resultant changes at their interface.

Among all BHJ materials, nonfullerene acceptors (NFAs) have become a pivotal area of research, greatly advancing the performance of bulk-heterojunction organic solar cells (OSCs).^3,28,29^ In recent years, numerous cutting-edge NFAs have been developed. Notably, the A-DA’D-A type acceptor, characterized by its ability to harvest near-infrared light and its high electron affinity, has achieved remarkable power conversion efficiencies (PCEs) exceeding 16% when combined with polymer donors in binary OSC systems.^30^ This success has inspired extensive research on optimizing the core structure of NFAs. Efforts include modifying alkyl chains, introducing halogen substitutions, extending the conjugation length, and altering the electron-deficient unit of the core.^31−33^

In parallel with advancements in NFAs, the development of wide band gap polymeric donors has become increasingly important for further enhancing the performance of OSCs, particularly in combination with NFAs. Pyrrolo[3,4-*c*]pyrrole-1,3(2H,5H)-dione (PPD)-based wide band gap polymeric donors have garnered attention due to their unique combination of electron-withdrawing imide groups and electron-rich pyrrole units, enabling tunable electronic properties and efficient charge transport.^34^ PPD-based polymers were initially developed for fullerene-based OSCs, where they demonstrated excellent performance owing to their large band gaps (∼1.90–2.20 eV) and strong absorption in the high-energy region of the solar spectrum.^35^ These polymers incorporate various electron-donating units such as benzodithiophene (BDT),^36^ dithienosilole,^37^ and cyclopentadithiophene,^38^ along with π-bridging units like thiophene^39^ and thieno[3,2-*b*]thiophene,^40^ resulting in versatile structural configurations. For example, PBDPD, a PPD-based polymer incorporating 4,8-bis(5-(2-ethylhexyl)thiophen-2-yl)benzo[1,2-b:4,5-b′]dithiophene (BDTT), achieved a PCE of ∼7% when paired with PC_70_BM, showcasing its potential as an efficient donor material.^34^

In this work, we focus on side-chain engineering of the donor materials, especially PPD-based wide band gap polymeric donors, since PPD-based polymers exhibit excellent photovoltaic performance along with PC_70_BM^41^ but have not yet been studied with the most promising and low-band gap NFA, namely the Y6-acceptor. Therefore, we synthesized a series of PPD-based polymers incorporating three different benzo[1,2-b:4,5-b′]dithiophene (BDT) derivatives including 4,8-bis(5-(2-ethylhexyl)thiophen-2-yl)benzo[1,2-b:4,5-b′]dithiophene [BDTTH], 4,8-bis(5-(2-ethylhexyl)-4-fluorothiophen-2-yl)benzo[1,2-b:4,5-b’]dithiophene [BDTTF], and 4,8-bis(5-(2-ethylhexyl)-4-chlorothiophen-2-yl)benzo[1,2-b:4,5-b’]dithiophene [BDTTCl]. The synthesized polymer series in this work aims to elucidate the role of halogen group substitutions in influencing the electronic properties, morphology, and overall device performance of the PPD-based polymeric donor in conjunction with the Y6 acceptor. This investigation into the binary system is crucial for understanding how specific chemical modifications, particularly introducing halogens, can impact the charge extraction, morphology, and efficiency of the device. In the first section of the study, a detailed examination of the binary system comprising the PPD-based polymeric donor and the Y6 acceptor is conducted. The second section of the study shifts focus to a ternary system, which involves the PM6 donor and Y6 acceptor, with the PPD-based polymers serving as an additive. This segment demonstrates the correlation of the insights from the binary system analysis with the current benchmark BHJ system of PM6:Y6. By examining the effects of the PPD-based polymers in both binary and ternary configurations, the study gives conclusions about the impact of chemical modifications on the performance and characteristics of OSCs.

## Results and Discussion

**Figure 1:**
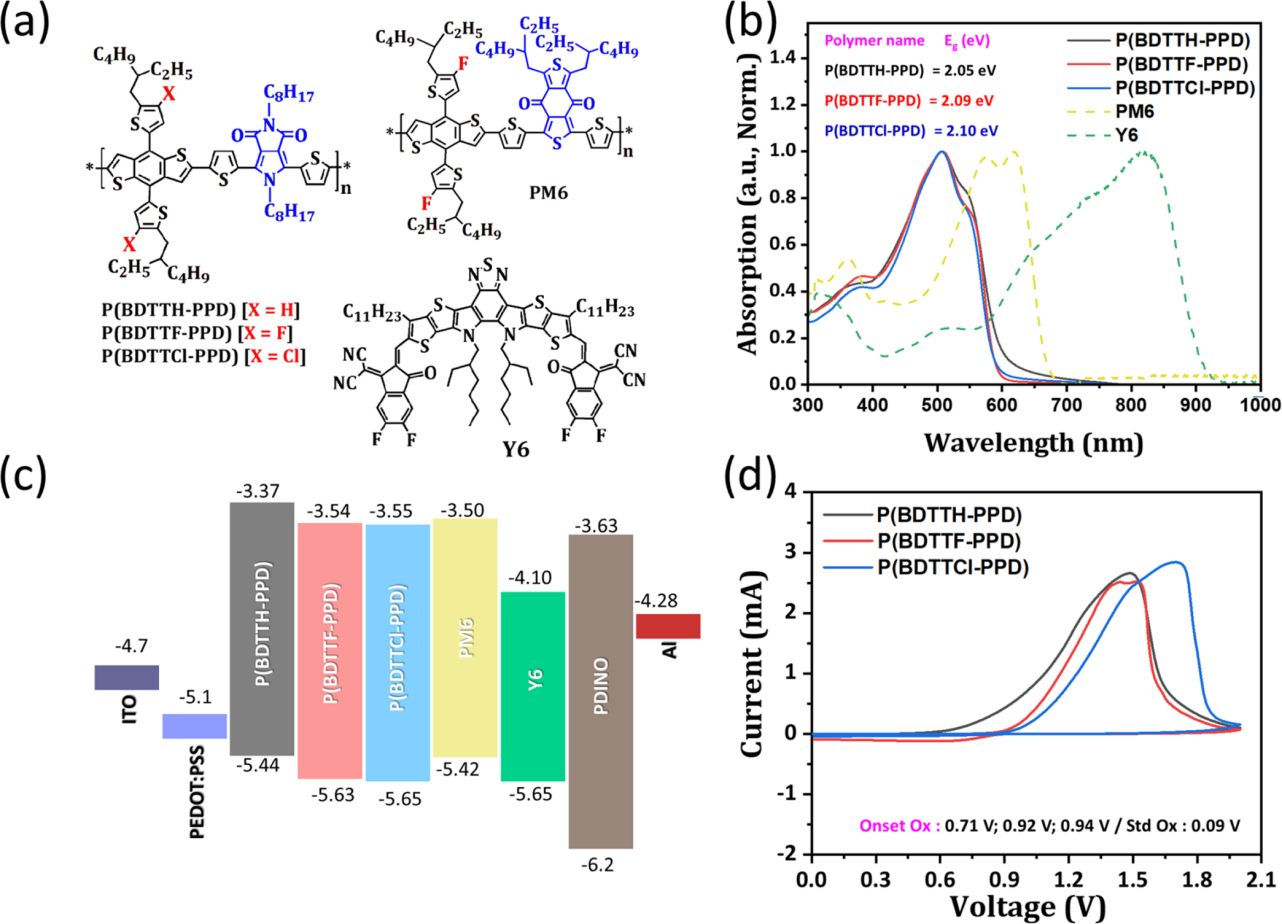
(a) Molecular structures of P(BDTTH-PPD), P(BDTTF-PPD), P(BDTTCl-PPD) and Y6, (b) Normalized UV–vis absorption spectra of thin films of the polymers, (c) Schematics of the active layer materials energy levels determined by cyclic voltammetry (CV), and (d) CV curves of P(BDTTH-PPD), P(BDTTF-PPD), P(BDTTCl-PPD).

Drawing inspiration from the halogen functionalization observed in PBDBT,^34^ we have synthesized polymers composed of a weakly electron-deficient 2,5-dioctyl-4,6-di(thiophen-2-yl)pyrrolo[3,4-*c*]pyrrole-1,3(2H,5H)- dione (PPD derivative) and three different electron-rich units including BDTTH, BDTTF, and BDTTCl. The chemical structures for the synthesized polymers such as P(BDTTH-PPD) and two of its halogenated derivatives, namely P(BDTTF-PPD) and P(BDTTCl-PPD), which incorporate fluorine (F) and chlorine (Cl) in BDT units, are presented in Figure 1a. The synthetic route to polymers, P(BDTTH-PPD), P(BDTTF-PPD), and P(BDTTCl-PPD), are outlined in Scheme S1. The NMR spectra of the compounds are included in Figures S1 and S2. The BDT units enhance the polymer’s charge carrier mobility, which is attributed to their planar structure and strong pi-pi stacking interactions,^42,43^ while the PPD units are notable for their robust light absorption at shorter wavelengths and well controlled wavey backbone.^44^ This synergistic combination endows P(BDTTX-PPD), where X = H, F, Cl, with a wide absorption spectrum and efficient charge transport–essential qualities for high-performance solar cells. These derivatives exhibit a broad absorbance range (Figure 1b), extending from the ultraviolet to 600 nm, effectively complementing the absorbance characteristics of Y6 acceptors. Additionally, the HOMO/LUMO energy levels of P(BDTTH-PPD), P(BDTTF-PPD), and P(BDTTCl-PPD) were determined to be −5.44/–3.37, −5.63/–3.54, and −5.65/–3.55 eV (Figure 1c), respectively, using cyclic voltammetry (CV) (Figure 1d). These results align with the expectation that introducing highly electron-withdrawing elements into molecules leads to deeper energy levels and a reduced *E*_loss_ when paired with the Y6 acceptor, which has HOMO/LUMO levels of −5.65/–4.1 eV. The energy levels of all components, including the transport layers and electrode materials, are illustrated in Figure 1c. This holistic approach to molecular design in P(BDTTX-PPD) derivatives paves the way for advanced materials in solar cell applications, offering improved compatibility and efficiency in organic photovoltaic systems.

**Figure 2:**
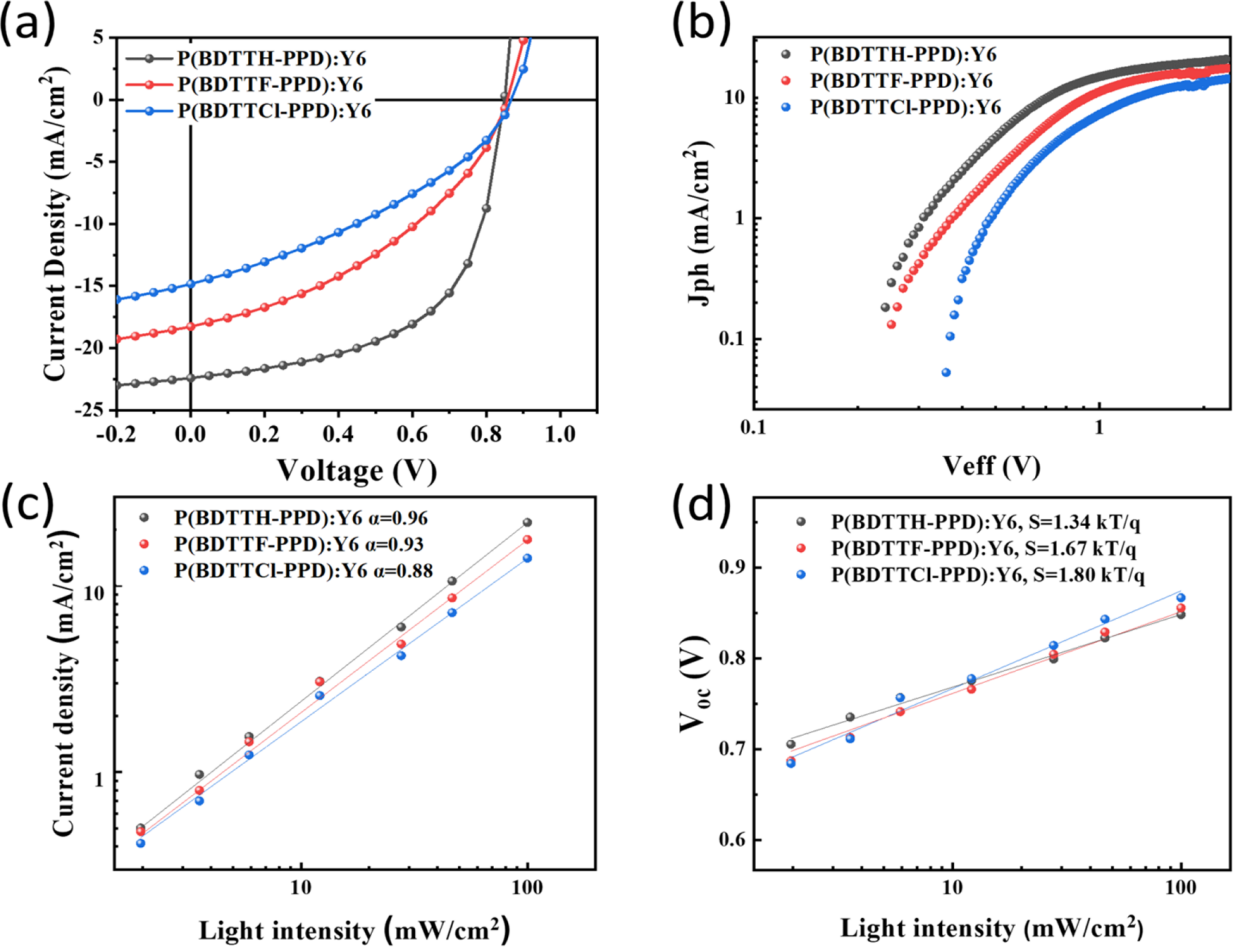
(a) *J–V* curves, (b) Photocurrent density (*J*_ph_) versus effective voltage (*V*_eff_), (c) dependence of *J*_sc_ on light intensity and (d) dependence of *V*_oc_ on light intensity.

To investigate the photovoltaic performance of the three new polymer donors with Y6 as acceptor, conventional device structures of ITO/PEDOT:PSS/active layer/PDINO/Al were fabricated and evaluated. As shown in Figure 2a, Table 1 and Figure S3, the P(BDTTH-PPD):Y6 blend demonstrates superior performance with a short-circuit current density (*J*_sc_) of 22.41 mA/cm^2^, suggesting an excellent capacity for photocurrent generation under irradiation. Although its *V*_oc_ of 0.85 V is not the highest among the devices, it still contributes to a robust power output. The fill factor (FF), at 58.2%, while not exceptional, indicates a reasonably quality solar cell. This is further evidenced by its power conversion efficiency (PCE) of 11.22%, which is the highest of the three devices, marking it as the most efficient in transforming absorbed light into electrical energy. In contrast, the P(BDTTF-PPD):Y6, despite having a slightly higher *V*_oc_ of 0.86 V, suffers from a lower *J*_sc_ of 18.27 mA/cm^2^ and a significantly reduced FF of 40.4%. These factors culminate in a PCE of 6.35%, positioning it as less efficient than P(BDTTH-PPD):Y6. Finally, P(BDTTCl-PPD):Y6, while boasting the highest *V*_oc_ at 0.87 V, falls behind in photocurrent generation with the lowest *J*_sc_ of 14.84 mA/cm^2^ and the lowest FF of 38.4%. These suboptimal parameters lead to the lowest overall efficiency, with a PCE of only 4.95%. Despite the advantageous high *V*_oc_, this device performance is hindered by its inability to effectively convert the generated current and voltage into power, as reflected in its low FF and PCE. Polymers substituted with F and Cl demonstrated deeper HOMO levels, which led to an improved *V*_oc_. In BHJ solar cells, *V*_oc_ is predominantly determined by the gap between the HOMO level of the electron donor and the LUMO level of the electron acceptor. Therefore, the deeper HOMO levels induced by F and Cl substitutions resulted in an increased gap, which in turn enhanced the *V*_oc_. Nonetheless, a higher *V*_oc_ does not invariably result in enhanced photovoltaic performance, i.e, despite the increase in *V*_oc_, the *J*_sc_ and FF, and consequently the PCE, have been negatively impacted. In the following sections, we will investigate into the underlying reasons for this compromise. The disparity in *J*_sc_ of OSCs was substantiated through IPCE measurements, where the P(BDTTF-PPD) and P(BDTTCl-PPD) based OSCs demonstrated significantly lower IPCE maximum values compared to those based on P(BDTTH-PPD), leading to a lower *J*_sc_ in the former in Figure S4.

**Table 1:** Photovoltaic Performance Parameters under AM1.5G (100 mW/cm^2^)^a^

	*J*_sc_ (mA/cm^2^)	*V*_oc_ (V)	FF (%)	PCE (%)
P(BDTTH-PPD):Y6	22.41(22.25 ± 0.4)	0.85(0.85 ± 0.005)	58.2(57.4 ± 0.8)	11.22(11.05 ± 0.4)
P(BDTTF-PPD):Y6	18.27(18.24 ± 0.3)	0.86(0.86 ± 0.004)	40.4(38.9 ± 0.9)	6.35(6.09 ± 0.2)
P(BDTTCl-PPD):Y6	14.84(14.65 ± 0.2)	0.87(0.87 ± 0.004)	38.4(35.8 ± 0.4)	4.95(4.55 ± 0.2)

a The average values are obtained from 20 individual devices.

Furthermore, to investigate the charge generation and the extraction properties in devices, we examined the exciton dissociation probability as shown in Figure 2b. The analysis focused on the photocurrent density (*J*_ph_) versus effective voltage (*V*_eff_) relationship, presented in a double-logarithmic graph. *J*_ph_ is defined as the difference between the current densities under irradiation (*J*_L_) and in the dark (*J*_D_). *V*_eff_ is calculated as the difference between the voltage at zero photocurrent density (*V*_0_) and the applied voltage (*V*).^45,46^ For all three polymer types studied, *J*_ph_ increased at lower *V*_eff_ and eventually plateaued, with a saturation photocurrent density (*J*_sat_) at a higher *V*_eff_, exceeding 3 V. This plateau suggests efficient sweeping out of photogenerated free charges.^46^ Under short-circuit conditions, the exciton dissociation probabilities (*P*_diss_*= J*_ph_/*J*_sat_) were 94.8, 90.8, and 87.6% for P(BDTTH-PPD), P(BDTTF-PPD), and P(BDTTCl-PPD), respectively. These results indicate efficient charge generation and collection of P(BDTTH-PPD),^22,27^ which is in agreement with our *J–V* results that, despite the halogenated polymers possessing a higher *V*_oc_, the FF and PCE are both lower.

Lastly, the light intensity (*P*_light_) dependent *J*_sc_ and *V*_oc_ are used to analyze the charge recombination properties within devices.^47,48^ As shown in Figure 2c, we analyzed how the *J*_sc_ varies with *P*_light_, expressed as *J*_sc_ ∝ (*P*_light_)^α^. The exponential factors (α) calculated for devices based on P(BDTTH-PPD), P(BDTTF-PPD), and P(BDTTCl-PPD) were 0.95, 0.92, and 0.87, respectively. These values suggest that the P(BDTTH-PPD) based device experiences a relatively higher efficiency in charge collection and minimal loss through nongeminate recombination compared to the others. As shown in Figure 2d, the relationship between the open-circuit voltage (*V*_oc_) and *P*_light_ follows the formula *V*_oc_ ∝ *S* (*kT*/*q*) ln(*P*_light_), where *k* is the Boltzmann constant, *T* is the temperature in Kelvin, and q is the elementary charge. The parameter *S*, which typically ranges between 1 and 2, indicates the extent of trap-assisted charge recombination within the device.^47,49^ The estimated values of S for devices based on P(BDTTH-PPD), P(BDTTF-PPD), and P(BDTTCl-PPD) are 1.34, 1.67, and 1.80, respectively, which reveals that P(BDTTH-PPD) device suppresses trap-assisted recombination in the device, leading to the better charge transport thus for a higher *J*_sc_ and FF. From these results, it can be inferred that the relatively smaller increase in *V*_oc_ for P(BDTTF-PPD) and P(BDTTCl-PPD)-based devices, despite their deeper HOMO levels compared to P(BDTTH-PPD), is due to the superior charge collection efficiency and minimized nongeminate recombination losses in P(BDTTH-PPD)-based devices.

The charge mobilities of devices are investigated using space charge limited current (SCLC) method (Figure S5 and Table S1). The P(BDTTH-PPD) device exhibits enhanced electron and hole mobility with a more balanced ratio μ_e_/μ_h_ which are responsible for the enhancement of FF and PCE.

**Figure 3:**
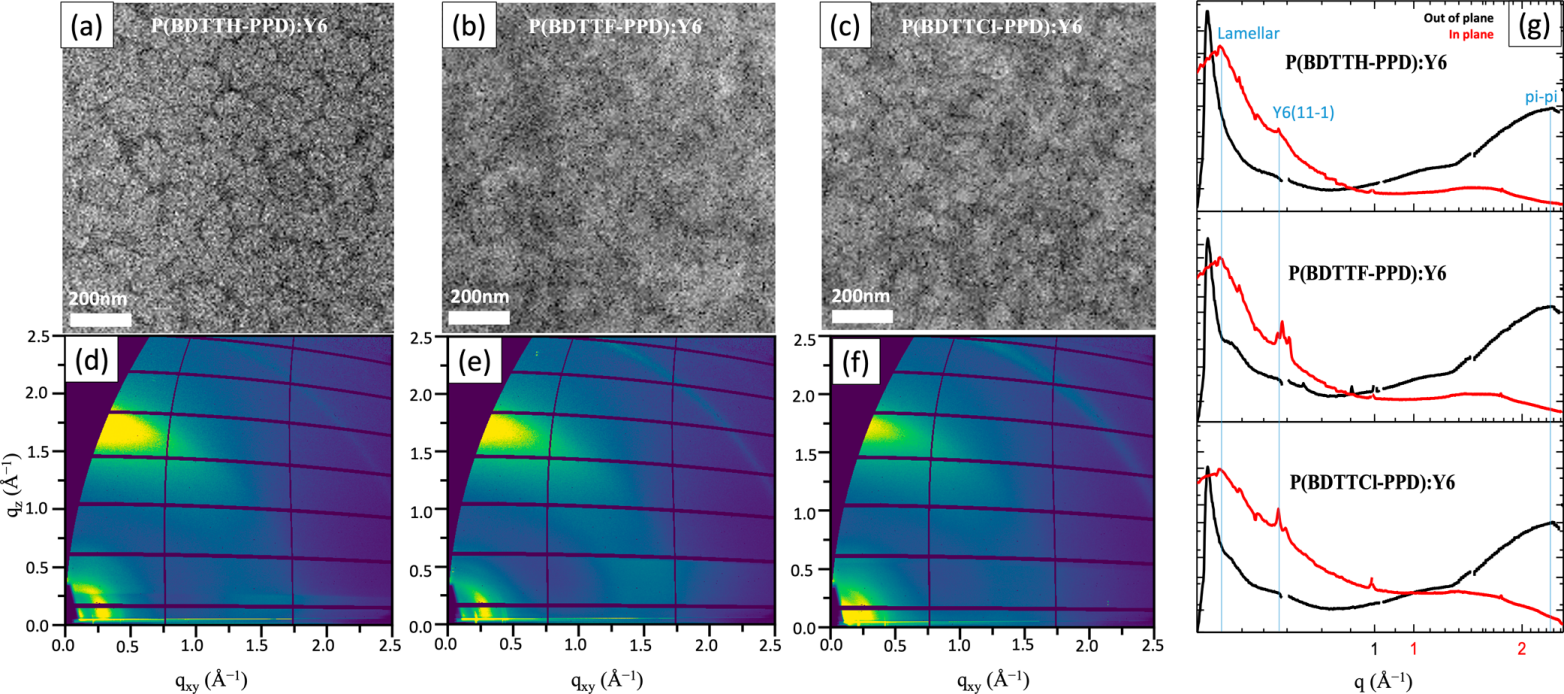
TEM images (a–c) of the binary BHJ film and the 2D GIWAXD data (d–g) of the binary BHJ films.

To gain deeper insight into why halogenation negatively impacts the optoelectronic properties, we conducted Transmission Electron Microscopy (TEM) analyses on P(BDTTH-PPD), P(BDTTF-PPD), and P(BDTTCl-PPD) devices blended with Y6. The morphologies of these blends are depicted in Figure 3a–c, respectively. The TEM images revealed that the P(BDTTH-PPD) (bright):Y6 (dark) blend demonstrated superior intermixing. Unlike the halogenated films (P(BDTTF-PPD) and P(BDTTCl-PPD)) with Y6, which showed ball-shaped aggregations, the P(BDTTH-PPD):Y6 blend exhibited an expanded width of Y6 fibers. This morphology included a well-developed nanoscale interpenetrating network and flower-like phase separations. These structural features are advantageous for enhancing charge carrier transport and reducing nongeminate recombination.^50,51^ This morphological superiority aligns well with the observed higher *J*_sc_ and FF in the P(BDTTH-PPD):Y6 devices. The structures of binary BHJ films were determined using grazing incidence wide-angle X-ray diffraction (GIWAXS), the results of which are shown in Figure 3d–g. It was observed that in the case of P(BDTTF-PPD):Y6 and P(BDTTCl-PPD):Y6 blends, the in-plane lamellar peak, (11-1) peak, and the out of plane π–π stacking peak, (11-1) peak, attributed to the Y6 crystalline structure, were prominently displayed. This observation can be attributed to the inclusion of F and Cl, which enhance interchain π–π stacking due to their high electronegativity, thereby increasing donor crystallinity.^23,24^ Additionally, the dipole–dipole interactions induced by halogen substitutions create a polar environment that hinders uniform blending with the nonpolar regions of Y6, ultimately promoting phase separation.^52^ This prominence is indicative of Y6 aggregation, as similarly observed in TEM analyses. In contrast, for the P(BDTTH-PPD):Y6 blend, the lamellar peaks were predominantly visible. The unique packing of the banana-shaped Y6 molecules allows for end group π–π stacking, facilitating the overlap necessary to form a polymer-like conjugated backbone. The lamellar packing is specifically assigned to the (110) lattice plane.^53^ Consequently, Y6 adopts a tilted molecular orientation, with the polymer-like backbone being inclined normal to the surface. This configuration is deemed more conducive to efficient charge transport. Therefore, the results suggest that the pronounced miscibility between P(BDTTH-PPD) and Y6, as corroborated by TEM findings, leads to a distinct, beneficial structural blend. The HOMO levels brought about by the F and Cl substitutions could potentially hinder miscibility and morphological synergy with the Y6 acceptor within the active layer, a factor crucial for the effective generation and transport of charge.

These complications may present themselves through a diminished *J*_sc_ and FF, ultimately impeding the device’s ability to generate photocurrent. We analyzed the optoelectronic properties and morphology of P(BDTTH-PPD), P(BDTTF-PPD), and P(BDTTCl-PPD) devices and observed that, despite the increased *V*_oc_ in halogenated devices due to reduced energy loss, there were notable issues in the blend morphology. While the *V*_oc_ improvement in halogenated devices is evident, the enhancement is smaller than expected given the deeper HOMO levels, suggesting that other factors, such as morphology, play a significant role. The integration with the nonfullerene acceptor Y6 was suboptimal, leading to microaggregations in the BHJ. These aggregations created defective interfaces and disrupted charge transport pathways. Consequently, these morphological imperfections resulted in FF and, subsequently, a decreased PCE in the devices. Our findings emphasize the nuanced impact that molecular architecture and intermolecular dynamics exert on the functional efficacy of photovoltaic, hence pointing to the need for an all-encompassing approach that harmonizes molecular design, miscibility, and morphology to collaboratively boost the photovoltaic attributes for the realization of high-efficiency solar cells.

**Figure 4:**
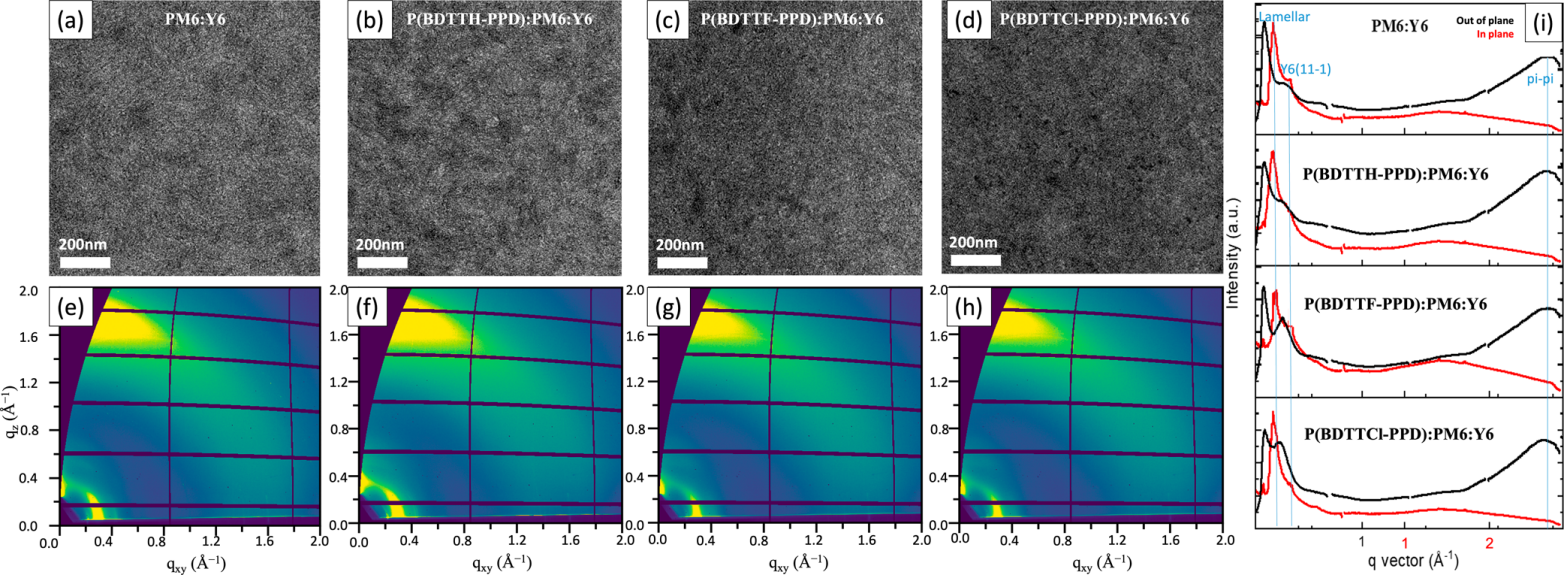
TEM images (a–d) of the ternary BHJ film and the 2D GIWAXD data (e–i) of the ternary BHJ films.

To further examine and apply the findings in the binary P(BDTTH-PPD):Y6 system to the benchmark PM6:Y6 system, we implement P(BDTTH-PPD) as solid-state additives to the PM6:Y6. As shown in Figure 4a–d, TEM images of PM6:Y6, PM6:Y6:P(BDTTH-PPD), PM6:Y6:P(BDTTF-PPD), and PM6:Y6:P(BDTTCl-PPD) are shown, respectively. Similar to the binary system where P(BDTTH-PPD) also shows better intermixing properties with Y6, the PM6:Y6 with P(BDTTH-PPD) shows very clear phase separation (flower-type donors and breached acceptors), and the halogenated ones are more amorphous, and the acceptors tend to form aggregations (ball shapes). To further explore the role of additive concentration on morphology, we examined PM6:Y6 blends with varying amounts of the P(BDTTH-PPD) additive (0.5, 1, 3, 5, and 10%) in Figure S6. At low concentrations (0.5–1%), the morphology exhibited fine fibril-like structures similar to the P(BDTTH-PPD):Y6 blend, with improved intermixing and nanoscale phase separation. However, at concentrations above 3%, excessively large fibril structures and microaggregates formed, disrupting the interpenetrating network. This morphology impacts the device performance as well (see below). The crystallinity of the ternary system was investigated by using GIWAXS in Figure 4e–i. Interestingly, the addition of P(BDTTF-PPD) and P(BDTTCl-PPD) to the PM6:Y6 blend resulted in the coexistence of lamellar peaks and (11-1) peaks. This observation suggests that these additives facilitate a mixed crystalline morphology and phase segregation within the blend, maintaining both lamellar and (11-1) crystalline orientations. These results are consistent with the TEM observations. Specifically, the substitution of halogen atoms in the polymer enhances interchain π–π stacking due to their high electronegativity, which reduces miscibility with the acceptor molecules. This leads to phase separation and the formation of spherical aggregates of Y6 acceptors, ultimately disrupting the interpenetrating networks required for efficient charge transfer. Such morphological disruptions impair charge transport and negatively impact overall device performance.^23−25,54^ However, the scenario altered significantly with the addition of P(BDTTH-PPD). In this blend, a notable decrease in the (11-1) peak was observed while the lamellar peaks were predominantly present, and the lamellar packing is assigned to the (110) lattice plane.^53^ This indicates that P(BDTTH-PPD) promotes a more uniform lamellar structure within the blend, potentially leading to an enhanced alignment of the molecular chains in a specific orientation. Such structural reorganization can be crucial for optimizing charge transport pathways, as it may favor a more efficient electron and hole mobility within the blend. These findings highlight the significant impact of solid additives on the crystalline morphology of PM6:Y6 blends, demonstrating their potential in tuning the photovoltaic properties of organic solar cells.^55−57^

**Figure 5:**
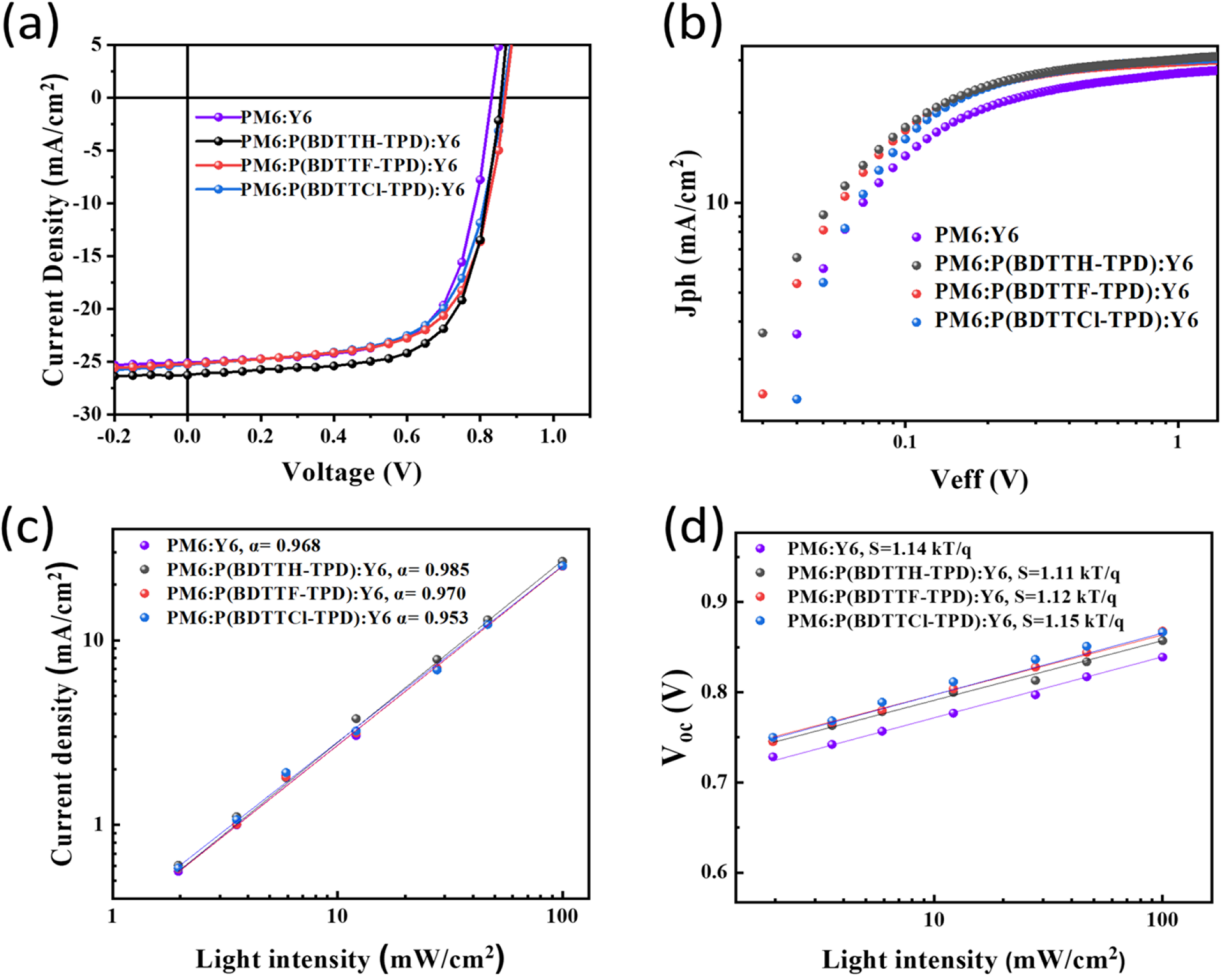
(a) *J–V* curves, (b) *J*_ph_ – *V*_eff_, characteristics, (c) dependence of *J*_sc_ on light intensity and (d) dependence of *V*_oc_ on light intensity.

As shown in Table 2, Figures 5a and S7, in examining the performance metrics of four distinct photovoltaic types, we observe varied strengths and efficiencies that cater to different aspects of solar energy conversion. In this study, we did not add other additives such as 1-chloronaphthalene (CN) and 1,8-diiodooctane (DIO) to see the effect of solid additives. The first type, PM6:Y6, establishes a baseline with a *J*_sc_ of 25.08 mA/cm^2^ and an *V*_oc_ of 0.84 V. Its FF at 67.0% and PCE of 14.12% depict a moderate performance, positioning it as a reference sample. Transitioning to the PM6:P(BDTTH-PPD):Y6 variant, we witness a notable improvement in performance, owing to the ideal phase separation and specific orientation. This type outperforms the others with the highest *J*_sc_ of 26.27 mA/cm^2^ and the highest FF of 68.4%, signaling its superior ability in current generation and efficient electricity conversion. Moreover, its PCE peaks at 15.45%, marking it as the most efficient cell. On the other hand, the PM6:P(BDTTF-PPD):Y6 cell type showcases its strength in voltage generation with the highest *V*_oc_ of 0.87 V, owing to the low *E*_loss_. However, suffering from the suboptimal phase intermixing, its *J*_sc_ and FF are slightly lower than the PM6:P(BDTTH-PPD):Y6, with a *J*_sc_ of 25.22 mA/cm^2^ and an FF of 66.7%. Its PCE still stands stronger than PM6:Y6 at 14.63%. This cell type might be preferred in situations where a slight trade-off in efficiency is acceptable for prioritized voltage value, i.e., photocatalysis, despite a slight compromise in other areas. Lastly, the PM6:P(BDTTCl-PPD):Y6 type presents a mixed performance. It has a *J*_sc_ of 25.28 mA/cm^2^ and a *V*_oc_ of 0.86 V, indicating competent current and voltage generation capabilities. However, its FF is the lowest at 64.9%, which slightly undermines its overall efficiency, reflected in its PCE of 14.11%.

**Table 2:** Photovoltaic Performance Parameters under AM1.5G (100 mW/cm^2^)^a^

	*J*_sc_ (mA/cm^2^)	*V*_oc_ (V)	FF (%)	PCE (%)
PM6:Y6	25.08(24.88 ± 0.2)	0.84(0.83 ± 0.006)	67.0(66.90 ± 0.7)	14.12(13.89 ± 0.1)
PM6:P(BDTTH-PPD):Y6	26.27(26.12 ± 0.1)	0.86(0.86 ± 0.005)	68.4(67.50 ± 0.6)	15.45(15.11 ± 0.1)
PM6:P(BDTTF-PPD):Y6	25.22(25.03 ± 0.2)	0.87(0.86 ± 0.007)	66.7(66.05 ± 1.0)	14.63(14.26 ± 0.1)
PM6:P(BDTTCl-PPD):Y6	25.28(25.14 ± 0.4)	0.86(0.86 ± 0.006)	64.9(63.62 ± 1.2)	14.11(13.68 ± 0.2)

a The average values are obtained from 20 individual devices.

The exciton dissociation probabilities under short-circuit conditions for the four photovoltaic PM6:Y6, PM6:Y6 with P(BDTTH-PPD), PM6:Y6 with P(BDTTF-PPD), and PM6:Y6 with P(BDTTCl-PPD) were 96.08, 98.95, 95.90, and 95.56%, respectively, as detailed in Figure 5b These results demonstrate efficient charge generation and collection with the largest voltage losses at open-circuit conditions in the PM6:Y6 with P(BDTTH-PPD) variant. Despite having a higher *V*_oc_, the devices with halogenated P(BDTTH-PPD) derivatives exhibit lower FF and PCE, aligning with earlier current–voltage results in Figure 2. Further investigation into the dependence of *J*_sc_ on light intensity, expressed as a proportionality to light intensity raised to a power (*J*_sc_ ∝ (*P*_light_)^α^), is presented in Figure 5c. The power factors calculated for the devices based on PM6:Y6, PM6:Y6 with P(BDTTH-PPD), PM6:Y6 with P(BDTTF-PPD), and PM6:Y6 with P(BDTTCl-PPD) are 0.96, 0.98, 0.97, and 0.95, respectively. These results suggest that P(BDTTH-PPD) into the PM6:Y6 system aids in charge separation and the P(BDTTF-PPD) and P(BDTTCl-PPD) variants pronounced nongeminate recombination. However, the altered morphology in the PM6:Y6 with P(BDTTCl-PPD) leads to a lower separation rate. Under open-circuit conditions, the degree of trap-assisted charge recombination within the devices is broadly similar in Figure 5d. The values for PM6:Y6, PM6:Y6 with P(BDTTH-PPD), PM6:Y6 with P(BDTTF-PPD), and PM6:Y6 with P(BDTTCl-PPD) are 1.14, 1.11, 1.12, and 1.15, respectively. This small variance indicates more severe trap disturbances in the devices with halogenated doped materials, a trend similar to the binary system shown in Figure 2c. As shown in Figure S8, compared with the reference devices (PM6:Y6), the devices based on the addition of P(BDTTH-PPD) showed an overall enhancement in the IPCE response across the whole absorption band of the active layer. Furthermore, similar to the binary system, the PM6:P(BDTTH-PPD):Y6 system exhibited faster charge mobility and a better balance of electron and hole charge transport in Figure S9 and Table S2. These findings highlight that device with halogenated blends experience more significant trap-assisted recombination compared to their counterparts, providing important insights for the design and efficiency optimization of these solar cell materials.

## Conclusions

In conclusion, this study explores the halogen substituent effect in OSCs, particularly focusing on PPD-based polymers with a Y6 acceptor, offering a nuanced understanding of the interplay between molecular design, device morphology, and photovoltaic performance. The integration of substituents, such as fluorine and chlorine, into the backbone of PPD-based polymers was found to significantly impact the optoelectronic properties and the morphology of the resultant bulk heterojunction solar cells. The introduction of halogens effectively modified the HOMO/LUMO energy levels, which theoretically should enhance the *V*_oc_ but resulted in lower FF and PCE. This paradoxical outcome was traced to the morphology of the blends. TEM analyses revealed that nonhalogenated P(BDTTH-PPD) blended with Y6 exhibited better intermixing than its halogenated counterparts. Specifically, halogenated blends such as P(BDTTF-PPD) and P(BDTTCl-PPD) displayed suboptimal morphologies with ball shape aggregations that led to defective interfaces and impaired charge transport. The study also explored the characteristics of the ternary system incorporating PM6 and found that integrating a synthesized PPD-based polymers as a solid additive in the PM6:Y6 system resulted in diverse morphological and electronic outcomes. Notably, P(BDTTH-PPD) showed improved intermixing and phase separation properties, leading to enhanced *J*_sc_ and FF. Additionally, incorporating P(BDTTH-PPD) into the PM6:Y6 system improved the PCE from 14.12 to 15.45%. The study underscores the complexity of balancing molecular design to optimize solar cell performance and opens pathways for further research in molecular engineering and morphology optimization in organic solar cells.

## Supporting Information Available

Detailed experimental procedures and additional data (synthetic route to polymers; NMR spectrum; the box chart in distribution of solar cell performance parameters; space-charge-limited current (SCLC); mobilities table) (PDF)
