# Supplementary material for: Reinforcing Bulk Heterojunction Morphology through Side Chain-Engineered Pyrrolopyrrole-1,3-dione Polymeric Donors for Nonfullerene Organic Solar Cells
Source: ACS Appl Energy Mater. 2025 Jan 7;8(2):1220–9. doi: 10.1021/acsaem.4c02670 (PMC11775866; doi:10.1021/acsaem.4c02670)
Supplement: Supplementary file 1 — ae4c02670_si_001.pdf [file ae4c02670_si_001.pdf]

Supporting information

Reinforcing bulk heterojunction morphology through side-chain-engineered pyrrolopyrrole-1,3-dione polymeric donors for nonfullerene organic solar cells

Danbi Kim,[1,2,3] Vellaiappillai Tamilavan,[4] Chieh-Szu Huang,[2] Yang Lu,[2,3] Eunhye Yang,[4] Insoo Shin,[5] Hyun-Seock Yang,[1] Sung Heum Park,[*4] Samuel D. Stranks[*2,3] and Bo Ram Lee[*1]

[1]*School of Advanced Materials Science and Engineering, Sungkyunkwan University (SKKU), Suwon 16419, Republic of Korea.*

[2]*Department of Chemical Engineering and Biotechnology, University of Cambridge, West Cambridge Site, Philippa Fawcett Drive, Cambridge CB3 0AS, United Kingdom.*

[3]*Cavendish Laboratory, University of Cambridge, J. J. Thomson Avenue, Cambridge CB3 0HE, United Kingdom.*

[4]*Department of Physics, Pukyong National University, Busan 48513, Republic of Korea.*

[5] *Department of Chemical and Biomolecular Engineering, New York University Tandon School of Engineering, Brooklyn, New York 11201, United States.*

E-mail: spark@pknu.ac.kr, sds65@cam.ac.uk and brlee@skku.edu

Experimental Section

Synthesis of polymers

Poly[4,8-bis(5-(2-ethylhexyl)thiophen-2-yl)benzo[1,2-b:4,5-b']dithiophene-alt-2,5-dioctyl-4,6-di(thiophen-2-yl)pyrrolo[3,4-c]pyrrole-1,3(2H,5H)-dione) (P(BDTTH-PPD)): A flame dried three neck round bottom flask containing a solution of monomers Bis-TMT(BDTTH) (0.18 g, 0.20 mmol) and PPD (0.14 g, 0.20 mmol) in chlorobenzene (20 mL) was purged well with argon for 30 min. Subsequently, Pd_2dba_3 (14 mg) and $P(o\text{-}tol)_3$ (28 mg) were added and the entire mixture was heated under reflux in argon for 48 h. The solution was cooled to RT and the solution was added drop wise to methanol (250 mL) with constant stirring. The precipitated polymer was allowed to settle. The precipitates were filtered and washed with methanol (50 mL) and acetone (50 mL). The polymer was then subjected to Soxhlet extraction with methanol and acetone for 24 h. The resulting polymer was dissolved in chloroform (10 mL) and filtered. The polymer was again precipitated by adding methanol (250 mL). The precipitates were filtered and dried under vacuum to afford pure polymer **P(BDTTH-PPD)** as a brown solid. Yield: 0.19 g (86%).

Poly[4,8-Bis(5-(2-ethylhexyl)-4-fluorothiophen-2-yl)benzo[1,2-b:4,5-b']dithiophene-alt-2,5-dioctyl-4,6-di(thiophen-2-yl)pyrrolo[3,4-c]pyrrole-1,3(2H,5H)-dione) (P(BDTTF-PPD)): The similar synthetic procedure is used as we mentioned above for **P(BDTTH-PPD)**. The copolymerization of monomers Bis-TMT(BDTTF) (0.19 g, 0.20 mmol) and PPD (0.14 g, 0.20 mmol) and followed by purification to afford pure polymer **P(BDTTF-PPD)** as a brown solid. Yield: 0.19 g (84%).

Poly[4,8-Bis(5-(2-ethylhexyl)-4-chlorothiophen-2-yl)benzo[1,2-b:4,5-b']dithiophene-alt-2,5-dioctyl-4,6-di(thiophen-2-yl)pyrrolo[3,4-c]pyrrole-1,3(2H,5H)-dione) (P(BDTTCl-PPD)): The similar synthetic procedure is used as we mentioned above for **P(BDTTH-PPD)**. The copolymerization of monomers Bis-TMT(BDTTCl) (0.20 g, 0.20 mmol) and PPD (0.14 g, 0.20 mmol) and followed by purification to afford pure polymer **P(BDTTCl-PPD)** as a brown solid. Yield: 0.20 g (86%).

Materials and characterization

The monomer namely 4,6-bis(5-bromothiophen-2-yl)-2,5-dioctylpyrrolo[3,4-c]pyrrole-1,3(2H,5H)-dione (Bis-Br(DTPPD)) was synthesized according to literature.[34] Whereas, monomers namely 4,8-Bis(5-(2-ethylhexyl)-thiophen-2-yl)benzo[1,2-b:4,5-b']dithiophene-2,6-diyl)bis(trimethylstannane) (Bis-TMT(BDTTH)), 4,8-Bis(5-(2-ethylhexyl)-4-fluorothiophen-2-yl)benzo[1,2-b:4,5-b']dithiophene-2,6-diyl)bis(trimethylstannane) (Bis-TMT(BDTTF)), and 4,8-Bis(5-(2-ethylhexyl)-4-chlorothiophen-2-yl)benzo[1,2-b:4,5-b']dithiophene-2,6-diyl)bis(trimethylstannane) (Bis-TMT(BDTTCl)) were purchased from SunaTech Inc and used as it is received. Reagents and solvents were obtained from Sigma-Aldrich. The UV- Vis absorption spectra were measured by using a T70+ UV/VIS spectrometer. The cyclic voltammogram (CV) of the polymer film was recorded using CH Instruments Electrochemical Analyzer. The CV instrument was calibrated with the most common ferrocene/ferrocenium ion (Fc/Fc^+) standard. The platinum working electrode, platinum wire (counter electrode), and Ag/AgCl electrode (reference electrode) were immersed in a solution of acetonitrile and a supporting electrolyte (0.1 M tetrabutylammonium tetrafluoroborate, Bu_4NBF_4) were subjected to CV analysis. The current density-voltage (J-V) characteristics of organic solar cell (OSC) devices were obtained using a Keithley 2400 Source Measure Unit. The performance of the solar cells was evaluated using a solar simulator that conforms to the Air Mass 1.5 Global (AM 1.5 G) standard, with a light intensity of 1000 W/m^2. The incident photon-to-current conversion efficiencies (IPCEs) of the OSCs were assessed over the wavelength range of 300–1000 nm, utilizing a xenon lamp for illumination, and calibration was performed using a silicon reference cell. Transmission electron microscopy (TEM) was performed using a Hitachi H-7500. GIWAXS characterizations were performed in Diamond Light Source i07 beamline with 10keV X-ray energy and Pilatus 2M detector, under He flow. Sample distance calibration was conducted using LaB6 calibrant and DAWN software 'and data were plotted using in-house script (https://github.com/YangLU996/CambriGIXS)'.

Device Fabrication

Prepatterned ITO-coated glasses were cleaned by sequential cycles of sonication in soap water, deionized water, ethanol, acetone, and isopropanol for 15 min, respectively. After UV/ozone

treatment for 30 min, A hole-transporting PEDOT:PSS (Baytron PH) was filtered and spin-coated on top of the treated-ITO at 4500 rpm for 30 s and dried over 145 °C for 15 min. The active layer solutions were prepared in chloroform (a solution of 1:1 (wt/wt) PPD based polymer:Y6 or 1:1.2 (wt/wt) PM6:Y6 or 0.9:0.1:1.2 (wt/wt) PM6:PPD based-polymer:Y6 blends in chloroform), and spin-coated on the PEDOT:PSS-coated substrates in a glove-box under N_2 atmosphere at 2500 rpm for 30 s. Then, a methanolic N,N′-Bis(N,N-dimethylpropan-1-amine oxide)perylene-3,4,9,10-tetracarboxylic diamide (PDINO) solution was spin coated on the top of active layer with 3000 rpm for 30 s. Finally, the Al (100 nm) electrode was evaporated onto the active layer via thermal evaporation at ~3×10^{-6} Torr.

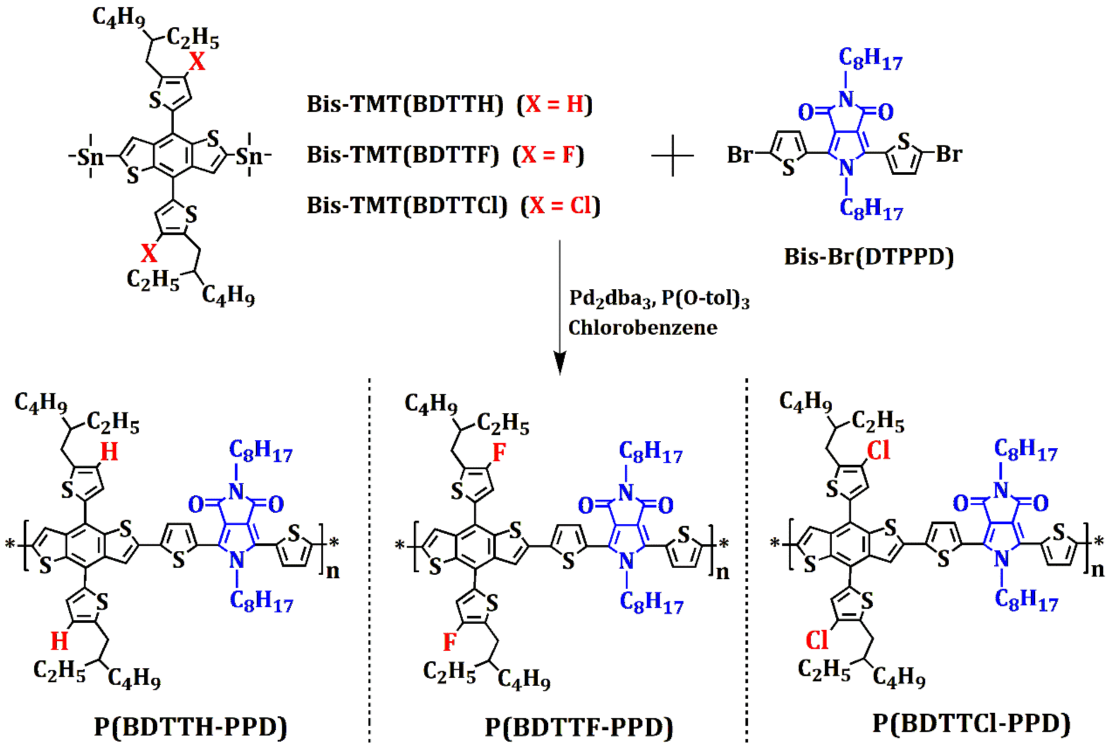

Scheme S1. Synthetic route to polymers.

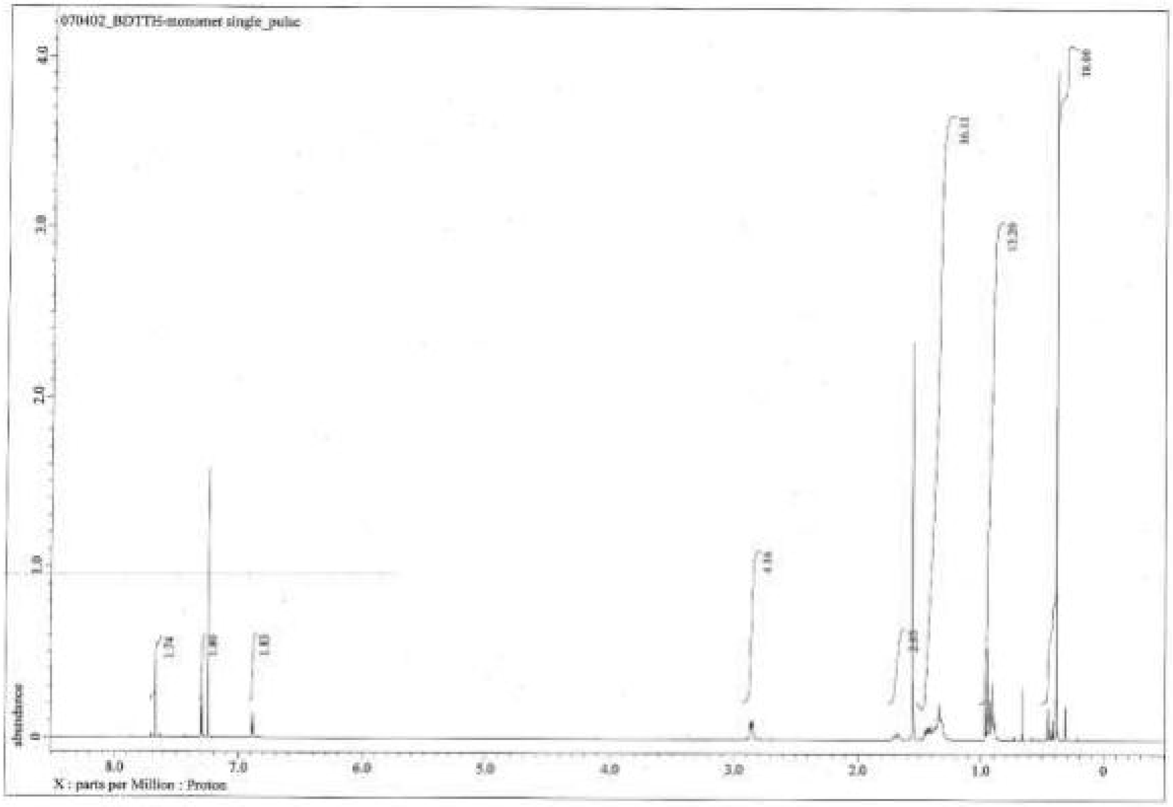

Fig. S1 ^{1}H NMR spectrum for BDTT.

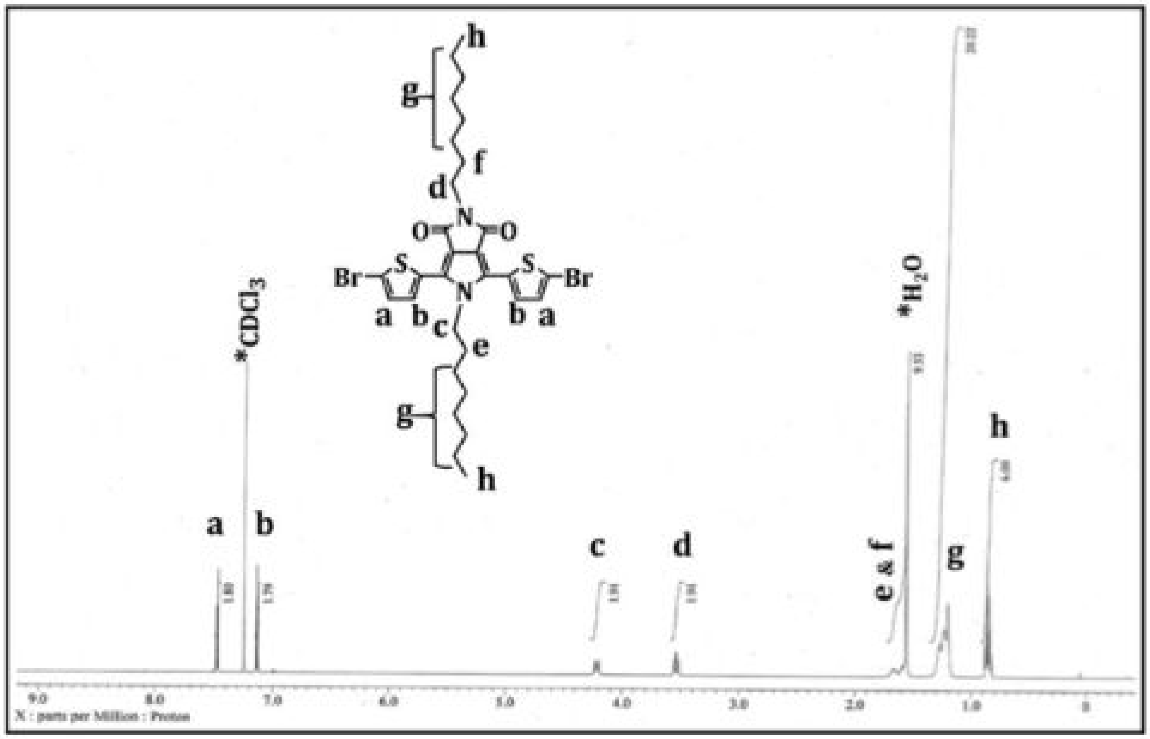

Fig. S2 ^{1}H NMR for Dibromo PPD.

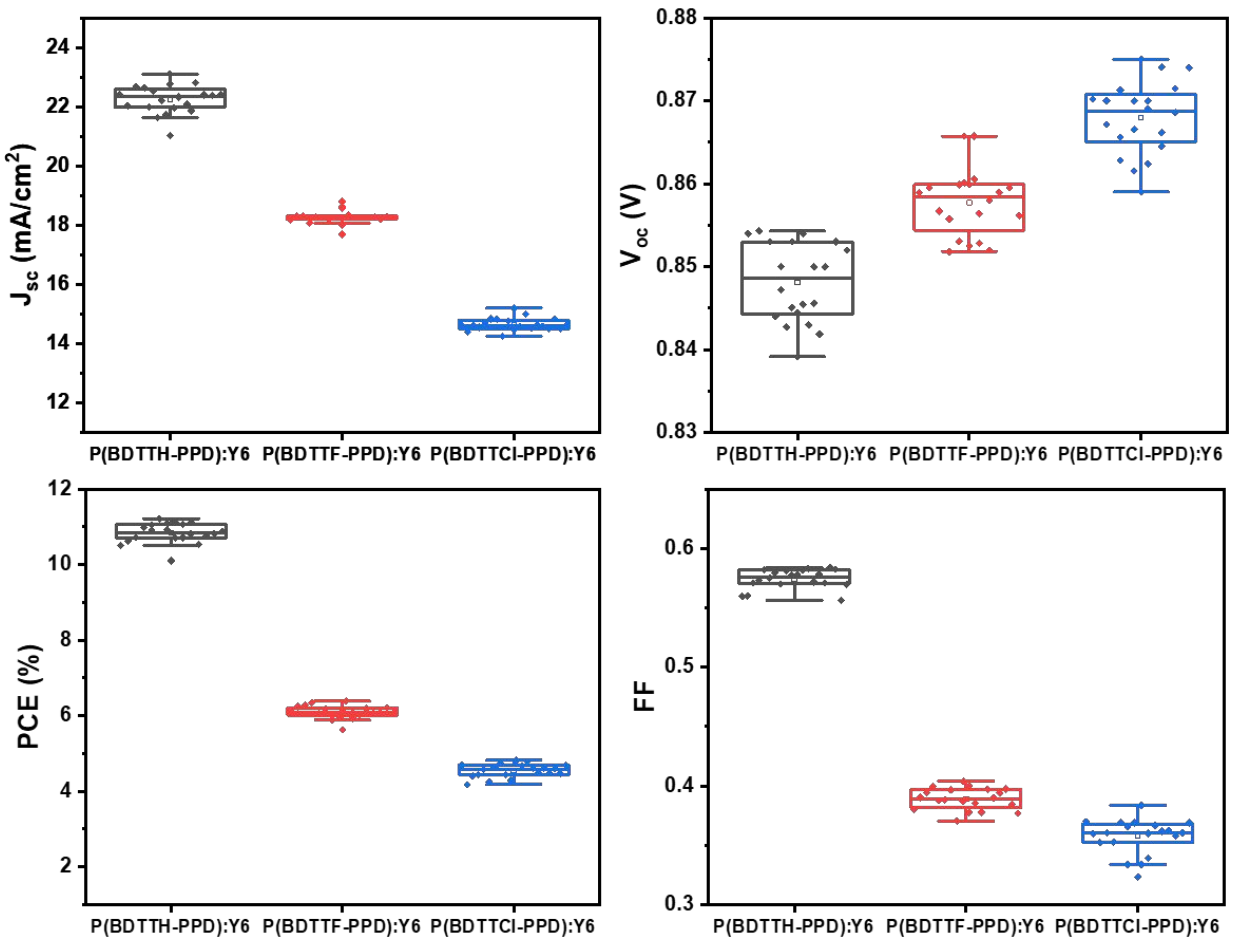

Fig. S3 The box chart in distribution of binary solar cell performance parameters (25~75% box limits with 1.5x interquartile range whiskers).

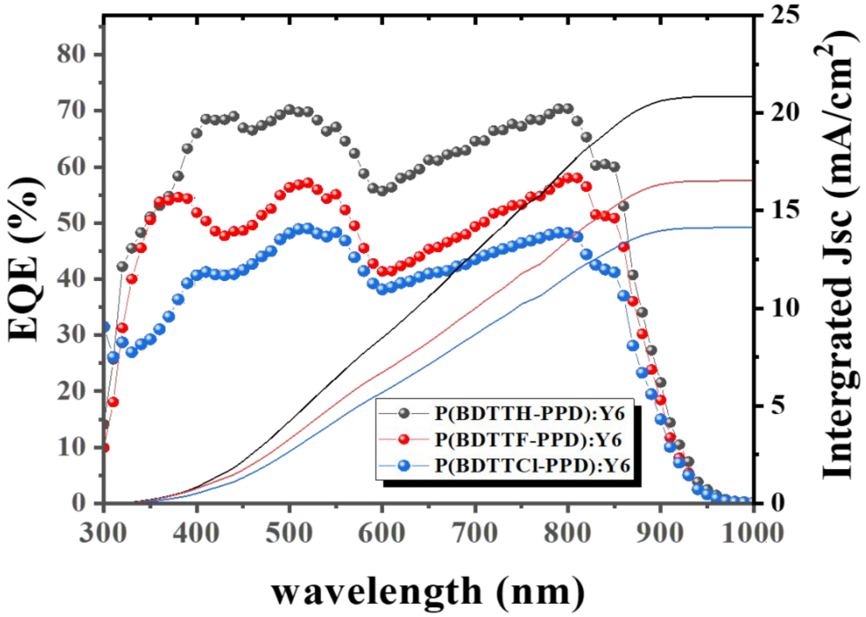

Fig. S4 The box chart in distribution of binary solar cell performance parameters of the binary OSCs

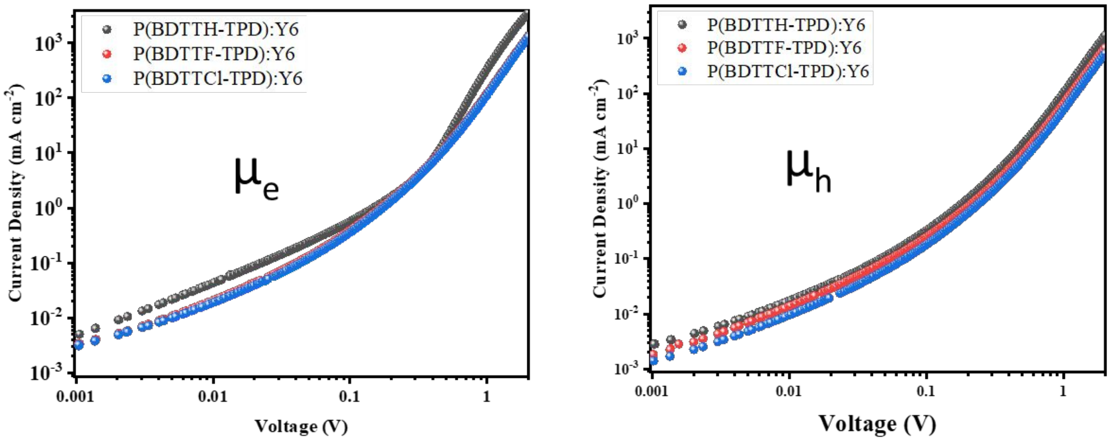

Fig. S5 Space-charge-limited current (SCLC) plots of the binary and ternary blend films with electron only device structure (μ_e) of ITO/ZnO/binary blend layer/PDINO/Al, hole only device structure (μ_h) of ITO/ PEDOT:PSS/binary blend layer/MoO_3/Ag

**Binary blend films**	μ_e	μ_h	μ_e /μ_h
P(BDTTH-PPD):Y6	4.52 * 10-4	3.28 * 10-4	1.38
P(BDTTF-PPD):Y6	3.95 * 10-4	2.43 * 10-4	1.63
P(BDTTCl-PPD):Y6	3.84 * 10-4	2.03 * 10-4	1.89

Table S1. Mobilities measured by SCLC method

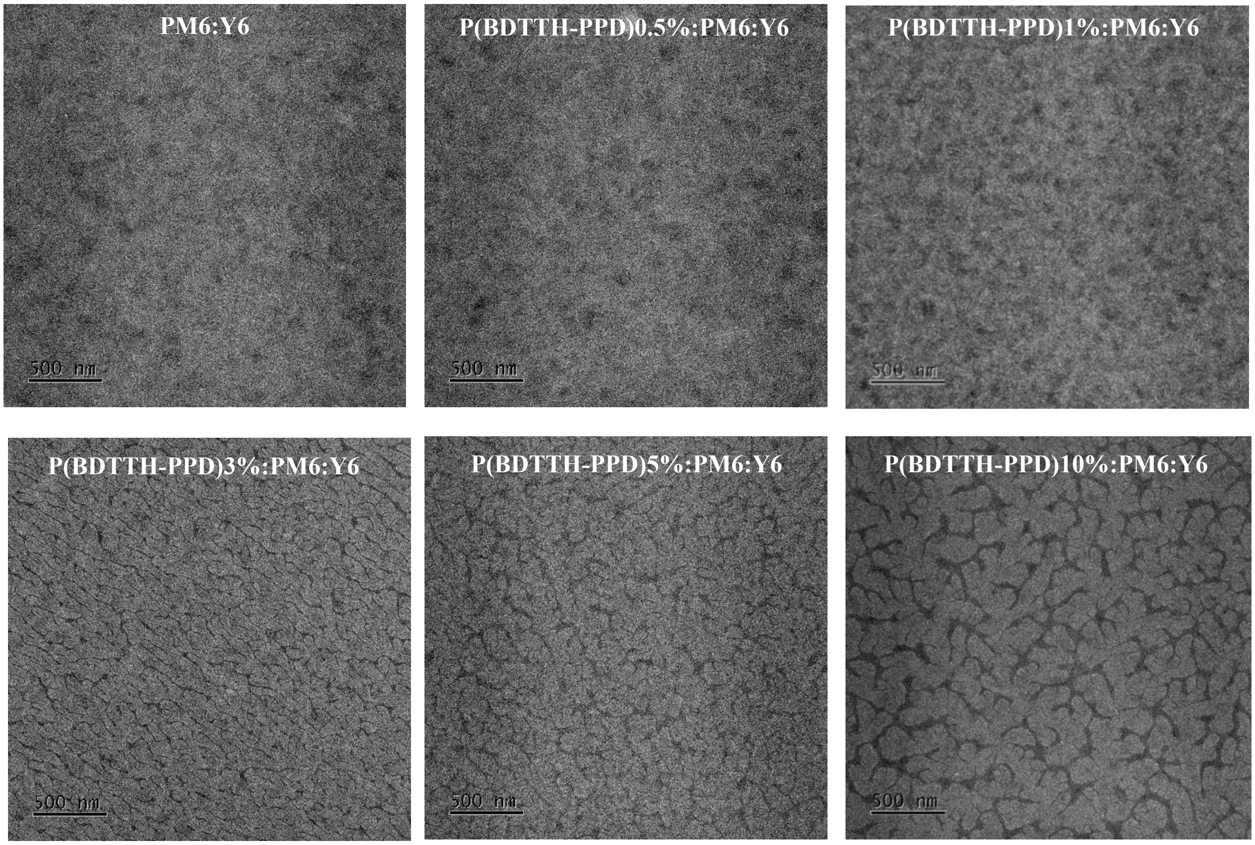

Fig. S6 TEM images with Concentration-Dependent Morphology of PM6:Y6 Blends with P(BDTTH-PPD) Additive

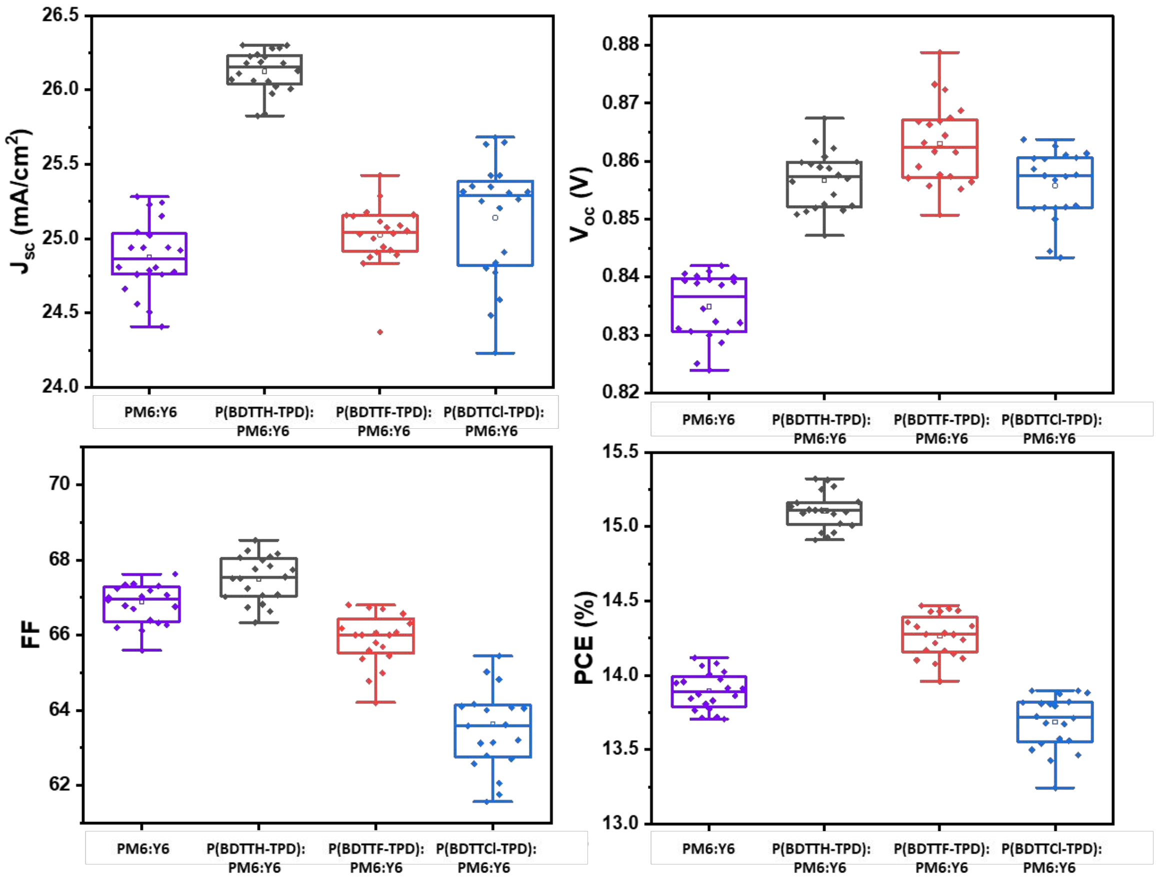

Fig. S7 The box chart in distribution of ternary solar cell performance parameters (25~75% box limits with 1.5x interquartile range whiskers).

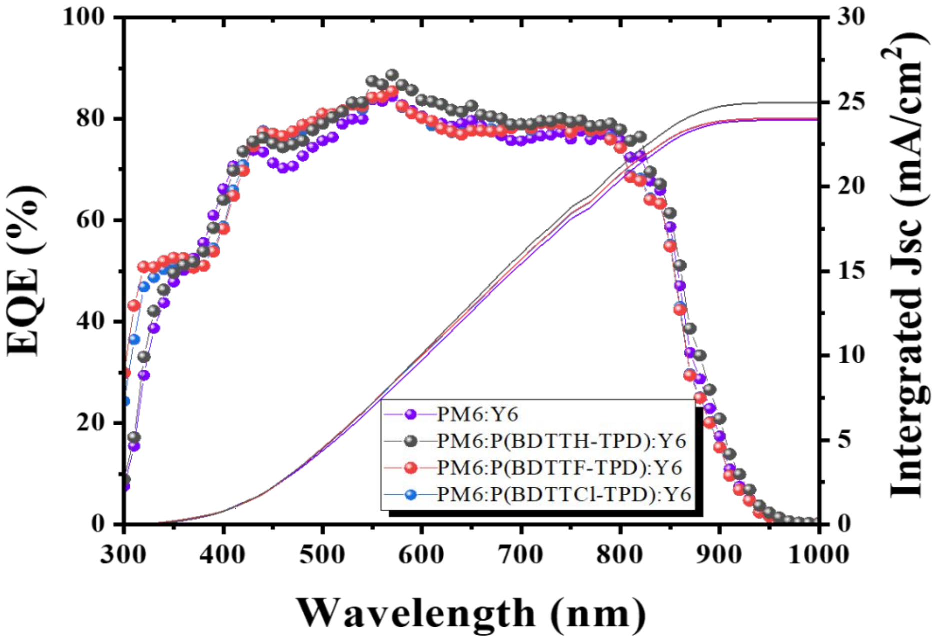

Fig. S8 The incident photon-to-current efficiencies (IPCEs) of the ternary OSCs.

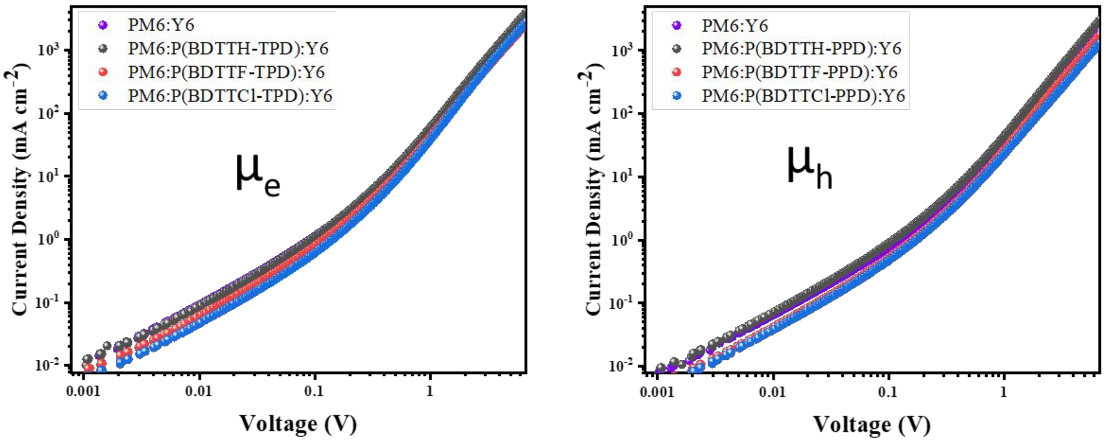

Fig. S9 Space-charge-limited current (SCLC) plots of the binary and ternary blend films with electron only device structure (μ_e) of ITO/ZnO/ ternary blend layer/PDINO/Al, hole only device structure (μ_h) of ITO/ PEDOT:PSS/ ternary blend layer/MoO_3/Ag.

**Ternary blend films**	μ_e	μ_h	μ_e /μ_h
PM6:Y6	$5.65 * 10^{-4}$	$4.63 * 10^{-4}$	1.22
PM6:P(BDTTH-PPD):Y6	$6.21 * 10^{-4}$	$5.42 * 10^{-4}$	1.15
PM6:P(BDTTF-PPD):Y6	$5.08 * 10^{-4}$	$4.12 * 10^{-4}$	1.23
PM6:P(BDTTCl-PPD):Y6	$4.86 * 10^{-4}$	$3.73 * 10^{-4}$	1.30

Table S2. Mobilities measured by SCLC method.
